# Nanomicelle formulation for topical delivery of cyclosporine A into the cornea: *in vitro* mechanism and *in vivo* permeation evaluation

**DOI:** 10.1038/srep12968

**Published:** 2015-08-26

**Authors:** Chuanlong Guo, Yan Zhang, Zhao Yang, Mengshuang Li, Fengjie Li, Fenghua Cui, Ting Liu, Weiyun Shi, Xianggen Wu

**Affiliations:** 1 State Key Laboratory Cultivation Base, Shandong Provincial Key Laboratory of Ophthalmology, Shandong Eye Institute, Shandong Academy of Medical Sciences, Qingdao 266071, China; 2 Qingdao Institute for Food and Drug Control, Qingdao 266071, China

## Abstract

A stable topical ophthalmic cyclosporine A (CsA) formulation with good tolerance and high efficacy is still a desire in pharmaceutics and clinics. This article describes the preparation of CsA containing nanomicelles using a polyvinyl caprolactam-polyvinyl acetate-polyethylene glycol (PVCL-PVA-PEG) graft copolymer. Both the polymer itself and the CsA nanomicelles were evaluated for cytotoxicity and ocular irritation. The *in vitro* uptake and intracellular fate of nanomicelles were characterized. *In vivo* cornea permeation test performed with 0.5 mg/mL CsA containing nanomicelles, and compared with a commercially available CsA (10 mg/mL) oil-based ophthalmic solution. The CsA nanomicelle ophthalmic solution was simple to prepare and remained storage stable. PVCL-PVA-PEG had no cytotoxicity as its monomer solution, and as its micelle solution (IC_50_(48 h) = 14.02 mg/mL). CsA nanomicelles also had excellent ocular tolerance in rabbits. The use of nanomicelles significantly improved *in vitro* cellular uptake, apparently by an energy dependent intracellular endocytosis pathway that involved early endosomes, late endosomes, lysosomes, and ER. *In vivo* permeation showed that 0.5 mg/mL CsA nanomicelles delivered high levels of CsA into the cornea, when compared to the oil-based 10 mg/mL CsA ophthalmic solution. These findings indicated PVCL-PVA-PEG nanomicelles could be a promising topical delivery system for ocular administration of CsA.

Cyclosporine A (CsA) is a potent immunosuppressant that is widely used to prevent corneal graft rejection and to treat autoimmune uveitis and dry eye syndrome. It is most commonly administered as an oil-based ophthalmic solution or as an eye drop emulsion (Restasis^®^), but unfortunately, these preparations are poorly tolerated and have side effects^1^,^2^,^3^. These two formulation types also result in low drug bioavailability because of the greater attraction of the drug to the lipophilic vehicle than to the more hydrophilic ocular tissues^4^. Thus, a need exists for a topical ophthalmic CsA formulation with good tolerance and high efficacy. Various attempts have been made to attain this goal of developing ophthalmic formulations of CsA, including the use of a cyclodextrin inclusion complex^5^,^6^, colloidal carriers (liposomes, nanoparticles, nanoemulsions, nanomicelles, and solid lipid nanoparticles)^4^,^7^,^8^,^9^,^10^,^11^,^12^, hydrogels^10^, and collagen shields^13^. Some of these systems have been shown to improve a number of the unfavorable physicochemical properties associated with CsA by enhancing its ocular availability and improving tolerance; however, none of these topical systems has yet reached the market. Furthermore, many other factors have contributed to failure to market a compound, such as high manufacturing costs, the use of harsh toxic solvents in the preparation process, toxicity issues, and stability problems. These factors have been particularly problematic for some of the colloidal carriers.

The use of polymeric nanomicelles is an attractive strategy for improving the corneal and conjunctival penetration of therapeutic drugs and peptides, for sustaining drug levels, and for reducing systemic side effects^14^. The present study examined the use of polyvinyl caprolactam-polyvinyl acetate-polyethylene glycol graft copolymer (PVCL-PVA-PEG, Soluplus^®^)-a new polymer with amphiphilic properties-for the formulation of nanomicelles for delivery of CsA in order to improve its tolerance and efficacy. The aim of this investigation was to ascertain whether a PVCL-PVA-PEG nanomicelle formulation could overcome some of the obstacles presented by topical ophthalmic CsA formulations and provide better ocular tolerance and cornea pharmacokinetic characteristics.

## Results

### Preparation and characterization of nanomicelles

**Figure 1:**
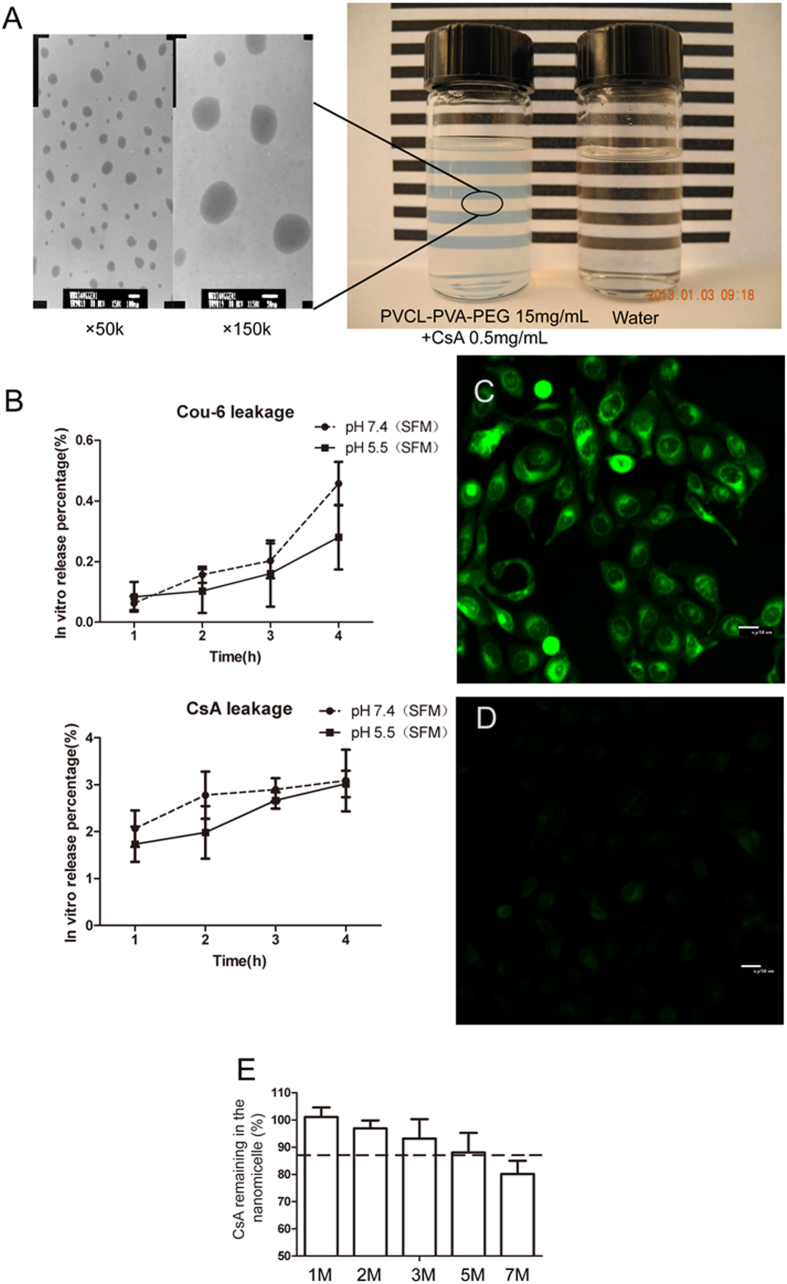
Characterization of the nanomicelle topical ophthalmic solution. (**A**) TEM morphology of nanomicelles (×50 k and ×150 k magnification) and the appearance of their ophthalmic solution. (**B**) Cou-6 and CsA leakage from nanomicelles. SFM with pH7.4 and pH5.5 was utilized as leakage mediums, respectively. (**C**) Confocal micrographs of HCECs cell monolayer incubated at 37 °C for 1 h with nanomicelles loaded with 0.45 mg/mL CsA or 0.05 mg/mL Cou-6. (**D**) Confocal micrographs of an HCECs cell monolayer incubated with free 0.05 mg/mL Cou-6 at 37 °C for 1 h. (**E**) Stability of CsA loaded nanomicelles. Bar = 50 μm.

The CsA nanomicelles were transparent, slightly opalescent, and slightly off-white when compared to water (Fig. 1A). Transmission electron microscope (TEM) analysis showed that the micelles were spherical or quasi-circular and homogenous, and no aggregates were present. No differences were observed between loaded and unloaded micelles in terms of morphology. The mean diameter, the Polydispersity index, and the Zeta potential of the CsA nanomicelles were 73.14 ± 24.42 nm, 0.067, and −6.7 mV, respectively, while the values for the blank nanomicelles were 78.03 ± 19.31 nm, 0.221, and −2.4 mV, respectively. The particle size obtained by photo-correlation spectroscopy was the same as the size visualized by TEM. The encapsulation efficiency was 97.11 ± 2.92%. The addition of Cou-6 and the loading of Cou-6 had no significant influence on nanomicelle diameter, Polydispersity index, or Zeta potential, when compared to CsA nanomicelles (*P* > 0.05).

Infrared (IR) absorption spectrophotometry, differential scanning calorimetry (DSC), and X-Ray diffraction (XRD) measurements indicated the absence of any free crystalline CsA in the nanomicelle preparation and that the polymer could inhibit drug crystallization during nanomicelle formation (Fig. S1, S2 and S3).

### *In vitro* leakage

Oil-based CsA leakage measurements revealed that less than 0.5% of the total Cou-6 and 4% of the total CsA leaked from the nanomicelles at pH 7.4 or pH 5.5, respectively, over a 4 h time course (Fig. 1B), suggesting that the loading of Cou-6 and CsA into the nanomicelles was stable even in a gradually acidifying intracellular environment. The human corneal epithelial cells (HCECs) treated with Cou-6 loaded nanomicelles showed significantly greater fluorescence intensity when compared to cells treated with free Cou-6 (Fig. 1C,D).

### Stability characterizations

Storage conditions of 25 °C with light protection resulted in slow, time dependent leakage of CsA from the nanomicelles. After 3 months of storage, 93.17 ± 7.15% of the CsA remained encapsulated in the nanomicelles, while 80.19 ± 4.83% still remained after 7 months (Fig. 1E), indicating that the CsA nanomicelles were very stable and would remain stable for at least 3 months under these conditions.

### *In vitro* toxicity tests

#### Polymer cytotoxicity test

**Figure 2:**
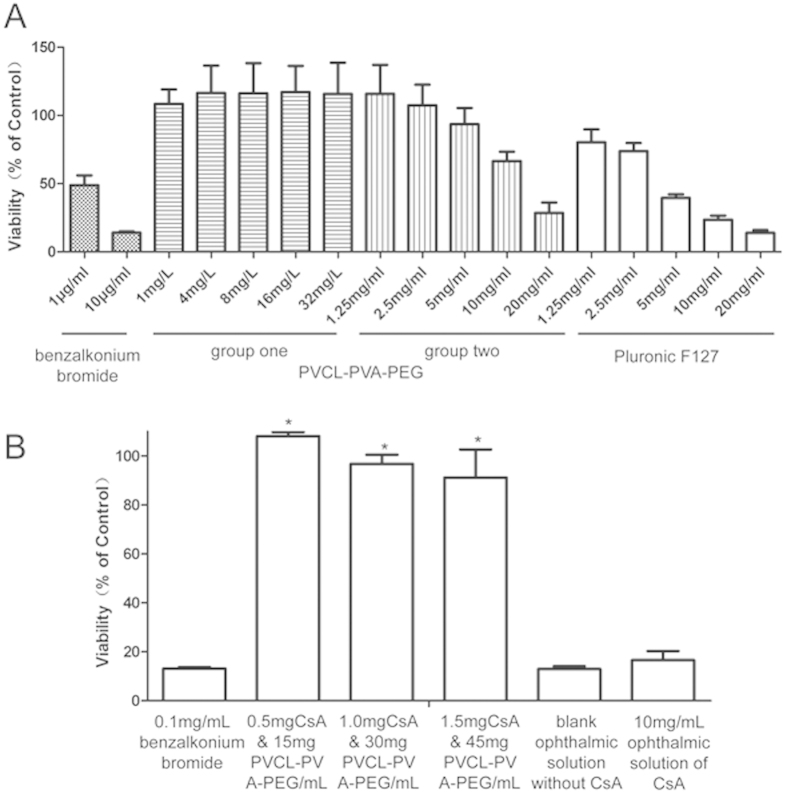
MTT results. (**A**) HCECs incubated with PVCL-PVA-PEG for 48 h; benzalkonium bromide and Pluronic F127 were used as controls (the concentration used in group one concentration was close to CMC, 7.8 mg/L). The toxicity of the non-assembled PVCL-PVA-PEG copolymer was also assessed. Group two was assessed at a higher concentration above the CMC (IC50 = 14.02 mg/mL in group two for PVCL-PVA-PEG and 4.28 mg/mL for F127, n = 3). (**B**) HCECs incubated for 4 h with CsA nanomicelles; benzalkonium bromide and commercial CsA eye drops were used as controls (*P < 0.05 when compared to the three benzalkonium bromide groups or two oil groups; n = 3).

The results of cell survival after treatment with PVCL-PVA-PEG are presented in Fig. 2A. The critical micellar concentration (CMC) of PVCL-PVA-PEG was approximately 8 μg/mL, so a concentration range from 1 μg/mL to 32 μg/mL was set for evaluation of cytotoxicity. After a 48 h incubation, no cytotoxicity was detected for the concentrations around the CMC of PVCL-PVA-PEG, and the cell survival rates were all approximately 100% for these five concentrations, when compared to the control. No cytotoxicity was observed, even at concentrations as high as 5 mg/mL. Slight cytotoxicity was noted when the concentration reached 10 mg/mL and 20 mg/mL. However, the cytotoxicity was much lower than that observed for benzalkonium bromide and Pluronic F127. Benzalkonium bromide (commonly used as a preservative in ophthalmic solutions in China, at an average concentration of 100 μg/mL) had significant cytotoxicity at levels as low as 1 μg/mL. Pluronic F127, even at a concentration of 1.25 mg/mL, killed approximately 20% of the cells after a 48 h incubation. The IC_50_ (48 h) was 14.02 mg/mL for PVCL-PVA-PEG and 4.28 mg/mL for Pluronic F127.

#### Cytotoxicity tests

The results for cell survival after treatment with nanomicelles are presented in Fig. 2B. After a 4 h incubation, no cytotoxic effects were noted for the CsA nanomicelles, even as the concentration of CsA nanomicelles was increased to two- and three-fold the concentration of the formulation used in subsequent *in vivo* animal studies. By contrast, the commercial oil-based CsA ophthalmic solution and the blank oil-based ophthalmic solution showed significant cytotoxicity. Benzalkonium bromide also showed significant cytotoxicity at a concentration of 0.1 mg/mL, which is the concentration commonly used in commercial ophthalmic solutions.

### *In vitro* uptake and intracellular fate in HCECs

**Figure 3:**
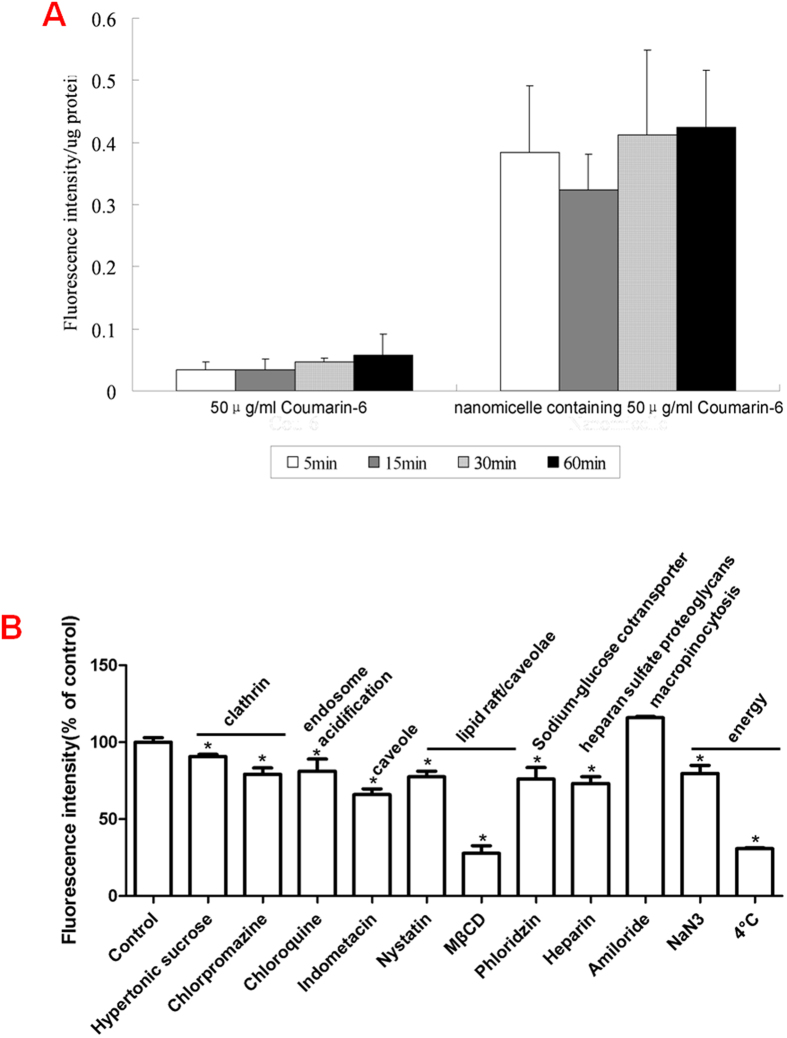
*In vitro* uptake characteristics. (**A**) Uptake of nanomicelles into HCECs. For easier detection, an additional 50 μg/mL Cou-6 was used compared to CsA nanomicelles (*P < 0.05 when compared with Cou-6; n = 3). (**B**) Endocytosis pathway analysis. Cells were pre-incubated for 30 min with the different inhibitors at the concentrations listed in Table S1, or uptake was performed at 4 °C or in the presence of NaN_3_. After pre-incubation, nanomicelles were added and incubated for another 1 h. The data are expressed as the fluorescence intensity (%) of negative controls (*P < 0.05 when compared with control group; n = 3).

As shown in Fig. 3A, the uptake of Cou-6 was significantly higher for the Cou-6 loaded nanomicelles than for free Cou-6 in solution at the indicated time points. Following a brief 5 min incubation, the uptake was recorded as 0.385 fluorescence intensity units per μg protein for the nanomicelles, which represented an 11.26-fold increase compared to the uptake of free Cou-6 (*P* < 0.05). The nanomicelles appeared to improve cellular uptake significantly. However, longer incubations did not result in further Cou-6 uptake in either group, indicating that the uptake of Cou-6 by the HCECs was time independent from 5 min to 60 min. The presence of Cou-6 in HCECs was also observed with confocal laser scanning microscopy (CLSM) at each time interval. The Cou-6 was mainly present in the cytoplasm in cells treated with free Cou-6 or nanomicelles, but the fluorescence intensity was much lower for the cells exposed to free Cou-6 in solution.

The energy dependence of nanomicelle uptake was investigated by evaluating the cellular uptake of nanomicelles at 4 °C or in the presence of a metabolic inhibitor (sodium azide). The nanomicelles were efficiently taken up by the cells incubated at 37 °C; however, when compared with the controls, the cellular uptake decreased significantly, by 69.25% at 4 °C or by 20.31% in the presence of sodium azide at 37 °C (Fig. 3B). This indicated an energy dependence and active trafficking of nanomicelles in the HCECs.

Different inhibitors of endocytosis were used to reveal the pathways involved in the uptake of nanomicelles by HCECs. Each inhibitor was first evaluated for its effects on cell viability with the 3-(4,5-dimethylthiazol-2-yl)-2,5-diphenyltetrazolium bromide (MTT) assay and none was found to cause any noticeable change in cell viability (data not shown). The cellular uptake of nanomicelles was inhibited to different extents following exposure to different inhibitors (Fig. 3B). Compared with the controls, inhibition was strongest for Methyl-β-Cyclodextrin (MβCD), which reduced the cellular uptake of nanomicelles by 72.17%. Hypertonic sucrose showed the least effect, with an inhibition of only 9.26%. The rank order of the other inhibitors was indomethacin (34.11%) > heparin (26.75%) > phloridzin (23.89%) > nystatin (22.38%) > chlorpromazine (20.83%) > chloroquine (18.86%). The macropinocytosis inhibitor amiloride had no effect on cellular uptake.

**Figure 4:**
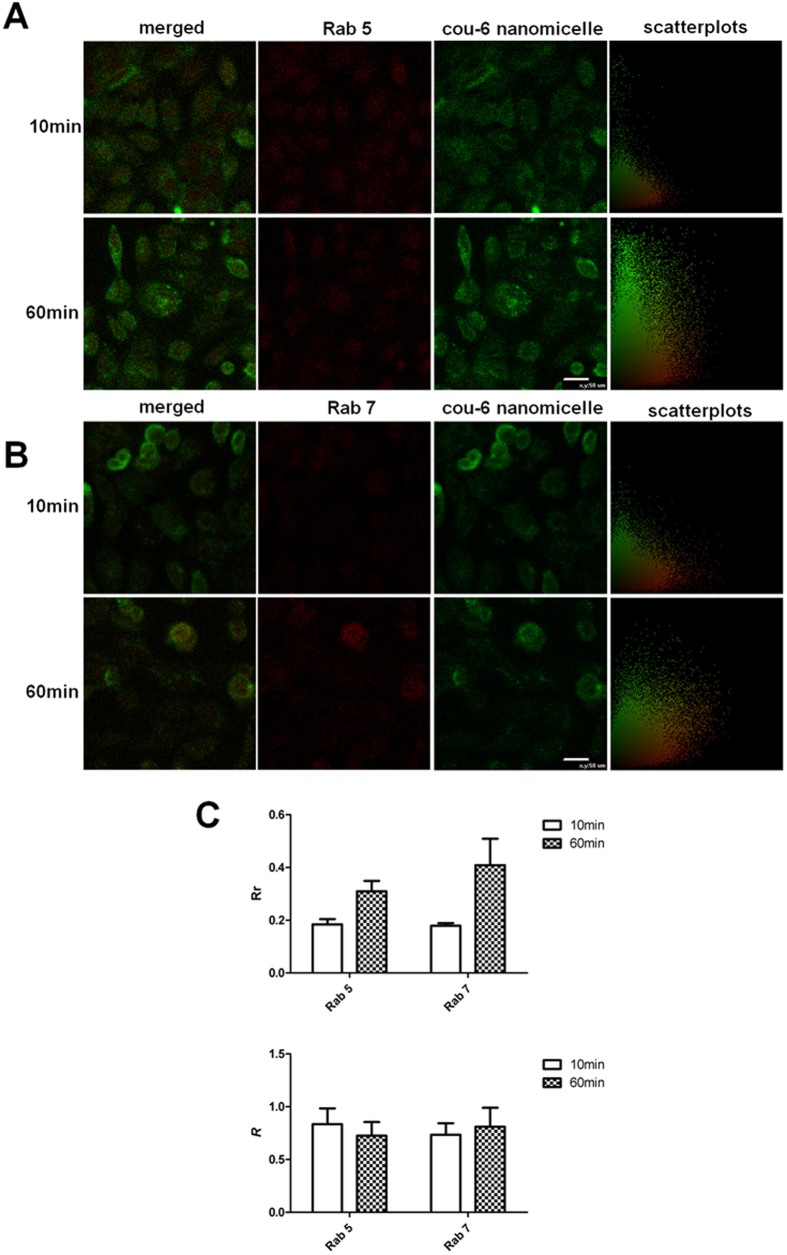
Intracellular trafficking in endosomes of nanomicelles. (**A**) Colocalization micrographs and scatterplots of nanomicelles with the early endosome marker Rab5 at 10 min and 60 min. (**B**) Colocalization micrographs and scatterplots of nanomicelles with the late endosome marker Rab7 at 10 min and 60 min. (**C**) Quantitative colocalization of nanomicelles with two endosomes markers by measuring Rr and *R* at 10 min and 60 min, respectively. Bar = 50 μm.

**Figure 5:**
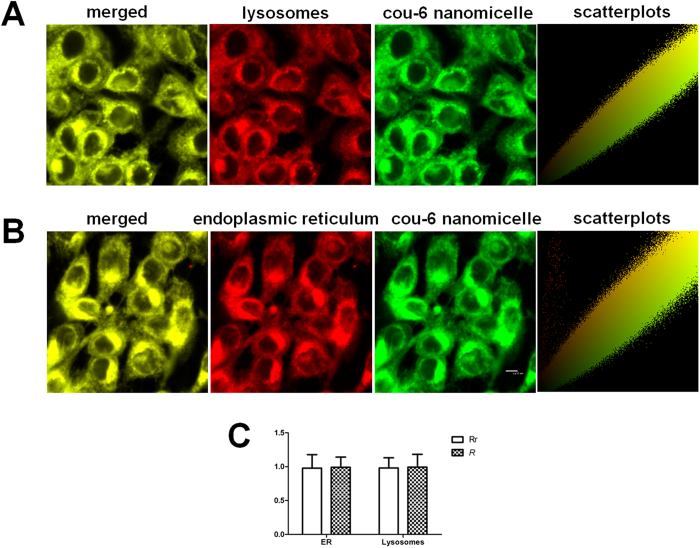
Intracellular trafficking of nanomicelles in lysosomes and endoplasmic reticulum (ER) after 1 h incubation at 37 °C. **(A**) Colocalization micrographs and scatterplots of nanomicelles with lysosomes. (**B**) Colocalization micrographs and scatterplots of nanomicelles with ER. (**C**) Comparison of quantitative colocalization of nanomicelles with lysosomes and ER by examining Rr and *R*. Bar = 12 μm.

Figure 4A,B show confocal micrographs of HCECs monolayers incubated at 37 °C for 10 min and 60 min, and reveal colocalization of Cou-6 containing nanomicelles with apical early endosomes (AEE) and late endosomes (LE). Additionally, by quantitative colocalization analysis and measuring Pearson’s correlation coefficient (Rr) and Mander’s overlap coefficient (*R*), the index of colocalization extent between image pairs, the intracellular trafficking of nanomicelles at different time was compared (Fig. 4C). Rr is used for describing the correlation of the intensity distributions between channels, while *R* indicating an actual overlap of the signals is considered to represent the true degree of colocalization. It was found the correlation of the intensity distributions between the AEE marker Rab 5 and nanomicelles improved from 10 min to 60 min incubation, as the Rr significantly improved (*P* < 0.05), and the similar result was also obtained for LE marker Rab 7 (*P* < 0.05). However, as Fig. 4C showed, all the Rrs were below 0.5, which indicated that the nanomicelles didn’t readily to just locate to AEE and LE at 10 min and 60 min, and the nanomicelles would be further transported to other subcellular organelles. The *R* in the AEE decreased with time but the decrease was not statistically significant (*P* > 0.05), while the *R* in the LE increased with time but the increase was not statistically significant (*P* > 0.05), and both the *R* for AEE and LE were higher than 70%. This results indicated that the nanomicelles were transported to the AEE with short time incubation (even less than 10min), and the nanomicelles in the AEE were quickly transported to the LE, but the LE was also a transit station, and the nanomicelles were further transported to other subcellular organelles. The intracellular location of Cou-6 containing nanomicelles was further localized to the lysosomes and endoplasmic reticulum (ER) by the application of lysosome- and ER-specific markers. The confocal micrographs of HCECs revealed colocalization of Cou-6 containing nanomicelles with lysosomes (Fig. 5A) and ER (Fig. 5B) after the incubation of Cou-6 containing nanomicelles at 37 °Cfor 60 min, revealing the actual transport of Cou-6 containing nanomicelles to lysosomes and ER. The quantitative measurements displayed that the Rr and *R* for both organelles were greater than 97% and 99% respectively (Fig. 5C), again supporting a lysosome- and ER-trafficking pathway for Cou-6 containing nanomicelles.

### *In vivo* cornea permeation characteristics

**Figure 6:**
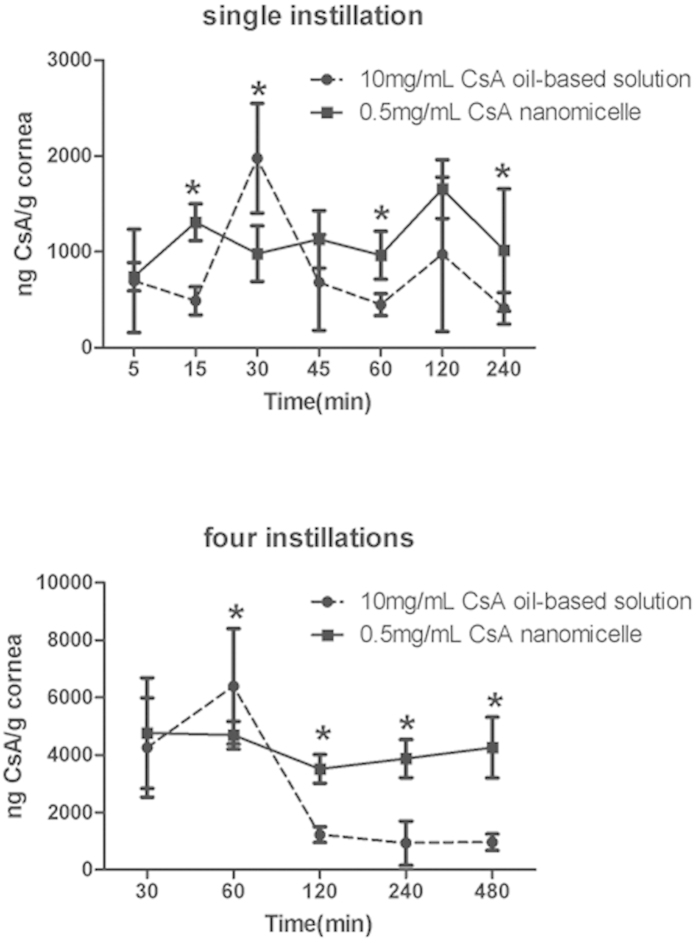
*In vivo* cornea permeation characteristics. CsA concentration in rabbit corneas after a single instillation (50 μL) and four instillations (50 μL/instillation at 10 min intervals) (*P < 0.05 when compared to 10 mg/mL CsA ophthalmic drop, n = 6).

The concentrations of CsA in the cornea following topical administration of the nanomicelles and the commercial formulation are shown in Fig. 6. The CsA levels of the nanomicelle groups were higher than those receiving the oil-based ophthalmic solution, and the difference was statistically significant (*P* < 0.05) at several time points, although the corneal concentrations of CsA at high dosages were only higher in the group treated with the oil-based ophthalmic solution at 30 min in the single instillation experiment and at 60 min with four instillations (*P* < 0.05).

CsA concentrations could not be determined in aqueous humor samples from any group, as they were less than 9 ng/mL. These results could not be shown graphically.

### Ocular tolerance

The modified Draize test revealed no eye irritation in the artificial tear groups, and no irritation was observed in eyes treated with PVCL-PVA-PEG at any concentration, although the median clinical scores showed significant differences compared to the artificial tear group at the 6 h observation time point (*P* < 0.05). The eyes treated with oil-based CsA ophthalmic solution showed only slight irritation at the 6 h observation time point, and the median clinical scores were significantly different when compared to the other five groups (Table 1).

## Discussion

In this study, CsA loaded PVCL-PVA-PEG nanomicelles were prepared using a simple solvent evaporation/film hydration method. A CsA dosage of 0.5mg/mL was used because it is a marketed dosage and was also an amount of CsA that could be easily encapsulated into the PVCL-PVA-PEG nanomicelles. The procedure was simple, and the obtained nanomicelles were well dispersed in aqueous solution with a narrow particle size distribution (Fig. 1A). The nanomicelle preparation did not require the use of organic solvents or other harsh toxic chemicals; only ethanol was used and it was evaporated during the preparation. Only moderate shaking was needed during the hydration. The non-encapsulated CsA was insoluble in the aqueous solution, so filtration through a 0.22 μm filter not only sterilized the preparation but also removed non-encapsulated CsA (although the encapsulation efficacy was high). These features would make this protocol easy to adapt to large-scale preparation if desired.

PVCL-PVA-PEG is a novel, FDA-approved polymer specifically designed for solid solutions^15^,^16^, and it was chosen for this investigation because of its aggregation behavior. At low concentrations, PVCL-PVA-PEG exists in solution as individual monomers, and thermodynamically stable micelles are formed as the copolymer concentration increases. PVCL-PVA-PEG has an extremely low CMC (6.61 × 10^−8^M)^17^, and it also has a low CMC in artificial tear solution (4.227 ± 0.317 mg/L, determined by a fluorescence probe technique using 1,6-diphenyl-1,3,5-hexatriene [DPH] as a fluorescence probe, unpublished data). By contrast, Pluronic F127 has a CMC = 2.80 × 10^−6^ M^18^, and its low CMC contributes to the stability of eye drop solutions for more than three months at 25 °C.

To our knowledge, this study is the first to explore the use of PVCL-PVA-PEG as an ocular drug delivery system. Therefore, the toxicity of this polymer must be adequately determined. In this investigation, we performed both *in vitro* and *in vivo* tests to evaluate the toxicity of PVCL-PVA-PEG in ocular drug delivery systems, and the results were promising. PVCL-PVA-PEG showed no cytotoxicity as a monomer (Fig. 2A). When the concentration was increased to 10 mg/mL, PVCL-PVA-PEG showed only slight cytotoxicity, evident as a decrease in cell viability. Its IC_50_ was 14.02 mg/mL, and only 15 mg/mL was used in the animal tests in this project. Pluronic F127 has been widely used and studied as an ocular drug delivery system, and it was selected as a control in this study. Although Pluronic F127 has an IC_50_ of 4.28 mg/mL (Fig. 2A), it is used at concentrations as high as 250 mg/mL^19^,^20^.

We also used the modified Draize test to determine *in vivo* effects. The PVCL-PVA-PEG solution, at concentrations as high as 45 mg/mL, did not cause irritation, although some clinical scores were evaluated at the 6 h time point (*P* > 0.05 when compared to the artificial tear group) (Table 1). Both the cytotoxicity and animal tolerance tests verified that PVCL-PVA-PEG was relatively safe for use as an ocular drug delivery system.

We also evaluated the PVCL-PVA-PEG nanomicelles after CsA loading for cytotoxicity and animal tolerance. The cytotoxicity testing, using 0.5 mg CsA and 15, 30, and 45 mg/mL PVCL-PVA-PEG, revealed no cytotoxicity, and no significant differences among these three polymer concentrations (Fig. 2B) (*P* > 0.05 when compared to each other). By contrast, the blank oil-based ophthalmic solution without CsA and 10 mg/mL oil-based CsA ophthalmic solutions caused significant cytotoxicity, with only approximately 20% cell survival after 4 h of incubation; this cytotoxicity was similar to that seen with benzalkonium bromide treatment (Fig. 2B) (*P* > 0.05 in the blank oil-based ophthalmic solution and 10 mg/mL oil-based CsA ophthalmic solution groups, compared to the benzalkonium bromide group). The cytotoxicity of these two oil-based solutions on HCECs might be partially attributed to the formation of a low permeable oil layer in the well, which hampered cell oxigenation, besides intrinsic toxicity. The animal tolerance test results were also consistent with the cytotoxicity responses, where only the 10 mg/mL oil-based CsA ophthalmic solution caused any (slight) irritation (Table 1). No severe irritation was observed in any of the tests, and these results seemed even less severe than those reported in some clinical studies^1^,^2^,^3^. One explanation might be that healthy rabbits were used in this test. Our rabbits did not have any eye diseases and showed good tolerance during the testing.

The oil-based CsA ophthalmic solution has been widely used clinically after cornea transplantation. These eyes might be more sensitive (particularly the corneas) after surgery, when they are still recovering. The nanomicelles consisting of 0.5 mg CsA and 15 mg PVCL-PVA-PEG/mL caused no irritation (Table 1), although some clinical scores were evaluated at the 6 h time point (*P* > 0.05 when compared to the artificial tear group) because an aqueous formulation is always preferable to an oil-based ophthalmic solution. In summary, all tests revealed that the PVCL-PVA-PEG and CsA nanomicelles should be safe for topical ocular application.

Although interest has been increasing in the development of nanomicelles for ocular drug delivery systems, no system has yet been successfully implemented despite decades of research. One of the main reasons for this failure is that the prepared micelles are unstable as aqueous dispersions under different conditions. In this research, for simplicity, the stability of the CsA nanomicelles was evaluated after storage in the dark at 25 °C (clear glass vials, light protected with aluminum foil), conditions that are easy to maintain in the topical ophthalmic solution industry. Under these storage conditions, only slow, time dependent leakage of CsA from the nanomicelles was observed (Fig. 1E); 93.17 ± 7.15% of the encapsulated CsA remained in the nanomicelles after 3 months of storage, which meets the limit set by the U.S. Pharmacopeia (proportion of the initial concentration remaining was 90%). Therefore, these CsA nanomicelles would be expected to remain stable for at least 3 months under these conditions.

We also examined leakage of encapsulated material from the nanomicelles as leakage could have invalidated our *in vitro* cell uptake analyses. Only a very low percentage of Cou-6 and CsA leaked from the nanomicelles (Fig. 1B), and free Cou-6 showed a much less intense fluorescence (Fig. 1C,D). This was consistent with the cell uptake test, suggesting that Cou-6 and CsA were stably loaded into the nanomicelles, and that the CsA or Cou-6 determined in cell uptake tests could be considered as encapsulated, and not arising from nanomicelle disassembly or leakage.

Uptake into corneal cells is essential for CsA accumulation and action in the cornea^21^. In this study, the cellular uptake was confirmed by partially replacing CsA with Cou-6, a fluorescent dye, so that the uptake could be monitored using a microplate reader, and directly observed by CLSM analysis^22^. After a 5 min incubation, the cell uptake of nanomicelles was 11.3-fold higher than that from a 50 μg/mL Cou-6 solution. Further incubation did not result in a significant increase in uptake in either group, so the mean cellular uptake reached a balance in 5 min (Fig. 3A).

The internalization of nanomicelles by HCECs was significantly reduced following incubation at 4 °C and in the presence of sodium azide (Fig. 3B), clearly indicating that uptake of the nanomicelles was an energy dependent process. Active endocytosis^23^,^24^,^25^ was involved, as determined by the reduction of the internalization of nanomicelles in HCECs following inhibitor treatments. The inhibitory effect of MβCD, a cholesterol depletion agent and an effective inhibitor of lipid raft/caveolae dependent endocytosis, was particularly strong compared to other inhibitors. Treatment with MβCD reduced the cellular uptake by 72.17% (Fig. 3B), indicating that the endocytosis of nanomicelles by HCECs involved lipid rafts in the cell membrane. The effects of nystatin were more moderate than those of MβCD, but the inhibition of cellular uptake of nanomicelles further confirmed the lipid raft dependency of endocytosis. The addition of hypertonic sucrose and chlorpromazine also caused a significant decrease in cellular internalization of nanomicelles compared to control cells (Fig. 3B), indicating a role for clathrin mediated endocytosis in the internalization of the nanomicelles.

Other endocytosis inhibitors, such as indomethacin, heparin, phloridzin, and chloroquine, also decreased the cellular internalization of nanomicelles (Fig. 3B), but amiloride had no effect (Fig. 3B), indicating that the internalization of nanomicelles was not mediated by macropinocytosis. We demonstrated that the nanomicelles could be endocytosed by HCECs via multiple pathways, involving both lipid raft and clathrin mechanisms but not macropinocytosis.

Colocalization of the endocytosed nanomicelles in HCECs was unable to reveal a precise intracellular pathway, but we could safely conclude that AEE, LE, lysosomes, and ER were all involved (Figs 4 and 5), similar to previous reports of nanoparticle intracellular trafficking in MDCK epithelial cells and Caco-2 epithelial cells^23^,^24^.

The most important parameter was the amount of CsA absorbed in the cornea upon instillation^4^. The cornea permeation of CsA was also examined *in vivo* following treatment with nanomicelles formulated from 0.5 mg CsA and 15 mg PVCL-PVA-PEG/mL. The low permeation of CsA after topical administration is usually overcome by clinical administration of CsA in a multi-dosage regimen to improve absorption into the cornea. We therefore compared the permeation of CsA using both single-instillation and four-instillation experiments. The pharmacokinetics in the cornea after a single instillation revealed that the CsA levels produced by nanomicelle administration were 6.32%–168.26% higher than those obtained by administering the oil-based ophthalmic solution (Fig. 6), and this difference was statistically significant (*P* < 0.05) at several time points (the exception was the 30 min time point, which showed that the pharmacokinetics of the oil-based ophthalmic solution were 101.28% higher those of the nanomicelles) (*P* < 0.05). The four-instillation regimen indicated that nanomicelle administration would have a significant advantage over administration of the oil-based ophthalmic solution, as the CsA levels were 11.94%-343.35% higher than those obtained using the oil-based ophthalmic solution (Fig. 6), and the difference was statistically significant (*P* < 0.05) at several time points (except for the 60 min time point, where the levels for the oil-based ophthalmic solution were 36.42% higher that those for the nanomicelles) (*P* < 0.05). Therefore, we conclude that the permeation efficacy was much greater for the nanomicelles than for the oil-based ophthalmic solution. The oil-based ophthalmic solution of CsA has been marketed with an indication for the prevention of corneal graft rejection, and the high efficacy observed following local treatment was consistent with the high levels of CsA found in the cornea^26^. We might boldly speculate that the CsA nanomicelles might be more effective in the prevention of corneal graft rejection, although this claim must be verified further with *in vivo* efficacy testing.

We failed to detect any CsA in any of our aqueous humor samples. Reports of the permeation of topical CsA into the aqueous humor have been controversial. For example, Yenice I *et al.* showed that the CsA concentrations in aqueous humor of animals could be determined only at low percentages (19.4% of aqueous humor samples) using CsA (0.1%) in castor oil-based ophthalmic solution, PCL/BKC nanospheres, or HA coated PCL/BKC nanospheres^27^. De Campos AM *et al.* reported negligible or undetectable CsA levels in inner ocular structures (i.e., iris/ciliary body and aqueous humor)^28^. Di Tommaso C *et al.* also obtained similar results with CsA using topical ocular delivery with micelle carriers^26^, whereas CsA has been detected successfully in some reports^8^,^29^. These contradictory results might have arisen because of the different formulation designs, animal testing protocols, and detection methods used in these reports. However, the common view is that topical formulations of CsA have shown efficacy in the treatment of extraocular diseases but have been inefficient at reaching intraocular targets.

Although the results presented here are promising, the following points require further investigation. The first concern remains safety. Indeed, PVCL-PVA-PEG is widely used in pharmaceutical formulations and is generally regarded as essentially a non-toxic and non-irritant material (Type IV DMF-23504), but a drawback of this polymer is its non-biodegradable property. Although some non-biodegradable polymers, such as Pluronic and PEGs, have already been successfully applied or researched as ocular topical drug delivery systems, the safety of their long-term use should be further investigated. The second concern is that the stability and shelf life of nanomicelles require further investigation. The third concern regards the cornea permeation characteristics with other dosage CsA loads in nanomicelles. In this manuscript, only 0.5 mg/mL CsA was used in the nanomicelles, and it quite successfully improved CsA penetration into the cornea. However, this concentration showed no significant advantages over commercial oil-based eye drops for delivery of CsA to aqueous humor. Therefore, a higher concentration of CsA in nanomicelles, even combined with another delivery system (e.g., mucoadhesive polysaccharide), should be designed and tested to improve *in vivo* permeation. The fourth concern is that permeation characteristics were only performed in healthy animals, and significant differences arise between the healthy and disease states. Therefore, the permeation characteristics in some animal models, such as cornea graft transplantation models, should also be investigated.

## Conclusions

The present study reported the preparation and physicochemical characterization of CsA nanomicelles using a novel polymer, PVCL-PVA-PEG. The cytotoxicity and cellular uptake were investigated, and the ocular irritation by the polymer solution and the nanomicelles was also evaluated. The *in vivo* cornea permeation of the CsA nanomicelles with single- and multiple-dosage instillations were evaluated. Overall, the nanomicelles were able to deliver high levels of CsA into the cornea, offering promise for local treatment of immune-mediated corneal disease. The CsA nanomicelles also showed excellent ocular tolerance in rabbit eyes and were very stable in storage as an ophthalmic solution. Therefore, these nanomicelles could represent a superior alternative to the currently applied oil-based CsA ophthalmic solution.

## Materials and Methods

### Materials and animals

Details of the materials and animal use information are described in the supporting information (SI) Materials and Methods. The animal care and procedures were conducted according to the Principles of Laboratory Animal Care. The use of animals in this study adhered to the ARVO Statement for the Use of Animals in Ophthalmic and Vision Research, and the animal study was approved by the Shandong Eye Institute Ethics Committee for Animal Experimentation (Approval document No 2012-6, Qingdao, Shandong, China).

### Preparation and characterization of the polymeric nanomicelles

Detailed methods describing the preparation and characterization of the polymeric nanomicelles are described in the SI Materials and Methods.

### *In vitro* CsA/Cou-6 leakage detection from nanomicelles

The protocol for estimating *in vitro* CsA/Cou-6 leakage from the nanomicelles is described in detail in the SI Materials and Methods.

### Stability Study

A physical stability study was performed to investigate the leakage of the drug from nanomicelles during storage. The CsA nanomicelle solution, sealed in 10 mL colorless glass vials, was stored in a thermostatically controlled container at 25 ± 2 °C and protected from light (packaged in aluminum foil). Samples were withdrawn at specific time intervals. The residual amount of the drug in the vesicles was determined after separation from unbound drug, as described previously for the separation of free drug^30^.

### Cell culture tests

HCECs were used in this study, and its culture procedures are described elsewhere in detail^31^.

### *In vitro* cytotoxicity test

The cytotoxicity of the PVCL-PVA-PEG polymer was tested on HCECs using two copolymer concentration ranges with standard MTT testing, as described elsewhere in detail^31^. Group one received 1, 4, 8, 16, and 32 mg/L polymer, and group two received 1.25, 2.50, 5.00, 10.00, and 20.00 mg/mL. The concentration range for group one was approximately the critical micellar concentration (CMC), and the toxicity of the non-assembled PVCL-PVA-PEG copolymer was assessed at this concentration range. The assembled PVCL-PVA-PEG copolymer was assessed in group two at concentrations higher than the CMC. Control cells were treated with PVCL-PVA-PEG (10 μg/mL), 1 μg/mL of benzalkonium bromide (a preservative widely used in ophthalmic solutions in China), or 1.25, 2.50, 5.00, 10.00, and 20.00 mg/mL Pluronic F127, a polymer widely used in ocular drug delivery systems. The experiments were performed in triplicate, with eight wells per measurement.

The cytotoxicity of the ophthalmic formulations was tested following a 4 h incubation; this duration was considered sufficient for observation of any toxic effects because ophthalmic solutions are rapidly cleared from the surface of the eye. Three concentrations of CsA PVCL-PVA-PEG nanomicelles were used: 0.5 mg CsA and 15 mg PVCL-PVA-PEG/mL; 1.0 mg CsA and 30 mg PVCL-PVA-PEG/mL; and 1.5 mg CsA and 45 mg PVCL-PVA-PEG/mL. Controls consisted of benzalkonium bromide (100 μg/ml), 10 mg/mL oil-based CsA ophthalmic solution, and blank oil-based ophthalmic solution without CsA.

### *In vitro* HCECs Uptake and mechanical characters

Uptake studies were conducted according to standard protocols^32^. Cou-6 fluorescence was measured at excitation-emission wavelengths of 465/502 nm. The uptake was normalized to the protein content of the cells, as measured by the Bradford assay (Beyotime BCA Protein Assay Kit, Beyotime Institute of Biotechnology). The results were reported as the mean fluorescence intensity per microgram of protein.

The endocytosis pathway of nanomicelles was investigated by the addition of specific inhibitors of particular cellular uptake pathways, as described in Table S1. HCECs seeded in a 12-well plate were pre-incubated with inhibitors for 30 min, followed by addition of Cou-6 containing nanomicelles and a further 1h incubation. A constant concentration of inhibitors was maintained during the nanomicelle incubation. After the incubation, cells were rinsed and the mean intracellular fluorescence intensity was measured by flow cytometry with excitation at 488 nm. The results are reported as the mean of the distribution of cell fluorescence intensity obtained by measuring ~10,000 cells. The energy dependence of uptake was determined by also performing the uptake at 4 °C or in the presence of NaN_3_.

Nanomicelles were detected in various types of endosomes by treating HCECs seeded on coverslips with a dispersion of 50 μg/mL Cou-6 containing nanomicelles in serum free medium (SFM) for 10 and 60 min. The cells were washed with PBS, fixed with 3.7% paraformaldehyde, incubated in PBS containing 0.1% Triton X-100 for 10 min, blocked with 5% BSA for 1 h, incubated with rabbit anti-Rab5 antibody and mouse anti-Rab7 antibody (1:500, Epotomics) at 37 °C for 2 h, and then treated with the matching fluorescently-labeled IgG in the dark for 1 h. The stained living cells were immediately detected by CLSM. The obtained fluorescence micrographs were further analyzed by Image pro plus 6.0 to obtain colocalization related parameters including Rr and *R*.

The location of nanomicelles in organelles was determined by staining HCECs in a glass-bottomed dish with 100nM Lyso-Tracker and 1μM ER-Tracker (Beyotime Institute of Biotechnology). The Cou-6 containing nanomicelles were added and incubated in the dark at 37 °C for 60 min. Cold PBS buffer was added to end the uptake and cells were incubated with SFM. The stained living cells were immediately detected by CLSM.

### *In vivo* cornea permeation

Rabbits were divided into four groups as follows: group 1 received a single instillation of oil-based CsA ophthalmic solution (10 mg/mL; 50 μL); group 2 received a single instillation of CsA nanomicelles (0.5 mg/mL; 50 μL); group 3 received four instillations of oil-based CsA ophthalmic solution (10 mg/mL; 200 μL); and group 4 received four instillations of CsA nanomicelles (0.5 mg/mL; 200 μL). In addition, four instillations of the formulations (200 μL) were administered to both eyes of the animals at 10 min intervals at 50 μL per instillation^27^. Six eyes of three animals were used for each time point in every group.

The animals were then sacrificed with a sodium pentobarbital overdose. Their aqueous humor was aspirated, and the corneas were collected at 5, 15, 30, 45, 60, 120, and 240 min following the instillation of the formulations in the single-instillation groups, and at 30, 60, 120, 240, and 480 min following the last instillation of the formulations in the four-instillation groups. All samples were stored at −80 °C until analysis.

### Quantitative determination of CsA in aqueous humor and corneas

Detailed methods describing the quantitative determination of CsA in aqueous humor and corneas are described in the SI Materials and Methods.

### *In vivo* ocular irritation tests

Ocular tolerance was tested using blank (no CsA) nanomicelles with copolymer concentrations of 15, 30, and 45 mg/mL, CsA loaded (0.05%) nanomicelles with a copolymer concentration of 15 mg/mL, sodium hyaluronate ophthalmic solution (1 mg/mL, HYCOSAN), and oil-based CsA (10 mg/mL) ophthalmic solution. Each compound was studied in six rabbits. Each formulation was instilled in the right eye for 30 minutes for a total of 13 times, leaving the left eye untouched as a control. Clinical signs were evaluated before the test and at 1, 6, and 24 h after the last instillation. The degree of eye irritation was scored using the modified Draize test^33^,^34^,^35^. Irritation was classified as one of four grades: practically non-irritating, score 0–3; slightly irritating, score 4–8; moderately irritating, score 9–12; and severely irritating (or corrosive), score 13–16^36^.

### Statistical analysis

The data were analyzed using SPSS software, version 11.5. MTT tests were analyzed by ANOVA with multiple comparisons, and comparisons of CsA in corneas between treated with CsA nanomicelles and commercial oil-based ophthalmic solution were determined using the rank sum test. The clinical scores for irritation testing were analyzed with nonparametric statistics, using the Kruskal-Wallis test. For all evaluations, a *P*-value less than 0.05 was considered statistically significant.

## Additional Information

**How to cite this article**: Guo, C. *et al.* Nanomicelle formulation for topical delivery of cyclosporine A into the cornea: *in vitro* mechanism and *in vivo* permeation evaluation. *Sci. Rep.* **5**, 12968; doi: 10.1038/srep12968 (2015).

## Supplementary Material

Supplementary Information

## Figures and Tables

**Table 1:** Median clinical scores (range) of irritation test (n = 6).

	1 h	6 h	24 h
Artificial tear	0 (0)	0 (0)	0 (0–1)
15 mg/mL PVCL-PVA-PEG	0 (0–1)	1.5 (0–3)*	0 (0–1)
30 mg/mL PVCL-PVA-PEG	0 (0)	1.5 (0–3)*	0 (0)
45 mg/mL PVCL-PVA-PEG	0 (0)	1 (0–3)*	0.5 (0–1)
0.5 mg/mL CsA Nanomicelle	0 (0)	1.5 (1–3)*	0.5 (0–2)
10 mg/mL CsA oil-based ophthalmic solution	3 (2–4)#	3.5 (2–6)#	2 (1–3)#

^*^Significant difference in median clinical scores compared to the artificial tear group (*P* < 0.05).

^#^Significant difference in median clinical scores compared to the other five groups (*P* < 0.05).
