# Supplementary material for: Nanomicelle formulation for topical delivery of cyclosporine A into the cornea: in vitro mechanism and in vivo permeation evaluation
Source: Sci Rep. 2015 Aug 26;5:12968. doi: 10.1038/srep12968 (PMC4549686; doi:10.1038/srep12968)
Supplement: Supplementary Information [file srep12968-s1.doc]

**Supporting Information (SI)**

**Nanomicelle formulation for topical delivery of cyclosporine A into the cornea：*in vitro* mechanism and *in vivo* permeation evaluation**

Chuanlong Guo^1,+^, Yan Zhang^2,+^, Zhao Yang^2^, Mengshuang Li^1^, Fengjie Li^1^, Fenghua Cui^1^, Ting Liu^1^, Weiyun Shi^1^, and Xianggen Wu^1,*^

1 State Key Laboratory Cultivation Base, Shandong Provincial Key Laboratory of Ophthalmology, Shandong Eye Institute, Shandong Academy of Medical Sciences, Qingdao 266071, China, 2 Qingdao Institute for Food and Drug Control, Qingdao 266071, China

* Correspondence: wuxianggen@126.com

+ these authors contributed equally to this work

**SI Materials and Methods**

**Chemical Reagents.** PVCL-PVA-PEG (Soluplus®) and Pluronic F127 were kindly donated by the BASF Corporation (Shanghai, China), and they were used as received. CsA, cyclosporine D (CsD), and blank oil-based ophthalmic solution without CsA were kindly provided by the North China Pharmaceutical Group New Drug Research and Development Co., Ltd. (Hebei, China). Oil-based CsA ophthalmic solution (30 mg/3 mL, North China Pharmaceutical Group New Drug Research and Development Co., Ltd. [Hebei, China]) was purchased from Qingdao Eye Hospital. Cou-6 and glucose were purchased from Sigma-Aldrich. Benzalkonium bromide solution (5%, Jiangxi Jingdong Pharmaceutical Ltd. [Jiangxi, China]) was used as the original solution and was diluted to the test concentration with cell culture media.

**Corneal epithelial cell line****.** Human corneal epithelial cells (HCECs; ATCC CRL-11135), which have been widely used in much corneal epithelial cell–related research, were kindly provided by Professor Chonn-Ki Joo (The Catholic University of Korea School of Medicine, Seoul, Republic of Korea). The cells were cultured in DMEM/F-12 (DF-12) medium with 10% fetal bovine serum (FBS; Gibco-BRL, Grand Island, NY, USA) under standard conditions (humidified atmosphere of 5% CO_2_ at 37 ℃).

**Animals.** New Zealand White rabbits were obtained from Qingdao Kangda Foodstuffs Co., Ltd., (Qingdao, Shandong, China; License No. SCXK [Lu] 20070023). The animal care and procedures were conducted according to the Principles of Laboratory Animal Care. All rabbits were healthy and free from clinically observable ocular abnormalities. The use of animals in this study adhered to the ARVO Statement for the Use of Animals in Ophthalmic and Vision Research, and the animal study was approved by the Shandong Eye Institute Ethics Committee for Animal Experimentation (Approval document No 2012-6, Qingdao, Shandong, China).

**Preparation and characterization of the polymeric nanomicelles**

***Preparation of nanomicelles***

Blank PVCL-PVA-PEG nanomicelles or CsA loaded PVCL-PVA-PEG nanomicelles were prepared by a solvent evaporation/film hydration method. Briefly, 150 mg of PVCL-PVA-PEG and 5.0 mg of CsA were co-dissolved in 2 mL of dehydrated ethanol in a round-bottomed flask. For the blank micelles, the CsA was omitted. The solvent was evaporated under reduced pressure at 40 ℃ to obtain a thin layer of uniform film on the wall of the flask. The residual film was then hydrated with 9.0 mL PBS (composition: Na_2_HPO_4_·12H_2_O 6.301 mM, NaH_2_PO_4_·2H_2_O 13.703 mM, at pH 6.5, adjusted to ~300 mOsmol/kg with glucose) under moderate shaking. Under these conditions, the amphiphilic PVCL-PVA-PEG copolymer self-assembled into nanomicelles, and the CsA was encapsulated within the micelles. The nanomicelles were filtered through a 0.22 μm filter to obtain sterile formulations^1^. After drug content analysis, the formulation was diluted with PBS to obtain a CsA concentration of 0.05%.

For MTT evaluation, CsA-loaded nanomicelles were obtained using the same procedure but with the addition of 10 mg of CsA and 300 mg of PVCL-PVA-PEG and of 15 mg of CsA and 450 mg of PVCL-PVA-PEG, resulting in 1 mg of CsA and 30 mg of PVCL-PVA-PEG/mL and 1.5 mg CsA and 45 mg PVCL-PVA-PEG/mL, respectively, in the nanomicelle solutions.

Coumarin-6 (Cou-6), a fluorescent probe^2^, was also similarly encapsulated into nanomicelles for measurements of *in vitro* cellular uptake and internalization evaluation. The nanomicelles were fabricated using 4.5 mg CsA and 0.5 mg Cou-6, resulting in 0.005% Cou-6 in the nanomicelle solution. Cou-6 was first dissolved in DMSO (the final concentration of DMSO in the incubation solution was 0.1%) and then was diluted with PBS to the test concentration of 0.005%, and this solution was used as the free Cou-6 solution group (control group) in the uptake test.

***Quantification methods of CsA/Cou-6***

The quantification of CsA was performed using a high-performance liquid chromatographic (HPLC) system fitted with a G1314A UV detector (detection at a maximum of 214 nm) and a G1311A Quat Pump (Agilent, US). Reverse-phase Agilent C_18_ columns (150 mm×4.60 mm, 5 μm; Agilent, US) were used for sample separation. The eluent for CsA consisted of 90% methanol and 10% water. The flow rate was kept constant at 1.0 mL/min and the column temperature was 50°C. The retention time of CsA was 6.6 min. Quantification of Cou-6 was also performed using an HPLC system fitted with a G1321A FLD detector (detection at Ex/Em=465/502 nm) and a G1311A Quat Pump (Agilent, US). Reverse-phase Agilent C_18_ columns (150 mm×4.60 mm, 5 μm; Agilent, US) were used for sample separation. The eluent for Cou-6 consisted of 90% methanol and 10% water. The flow rate was kept constant at 1.0 mL/min and the column temperature was 60 °C. The retention time of Cou-6 was 4.9 min.

***Size analysis and the zeta potential***

The mean particle size of the nanomicelles was determined by photo-correlation spectroscopy, using a Zetamaster (Malvern MS2000, UK) equipped with Malvern PCS software (version 1.27). The reading was performed at a 90° angle relative to the incident beam. Electrophoretic mobility was obtained with a laser Doppler anemometer, using the same instrument. The zeta potential value was calculated with the software using Smoluchowski’s equation.

***Morphological characterization***

Nanomicelles were observed and photographed with a transmission electron microscope (TEM, JEM-1200EX, JEOL Ltd., Tokyo, Japan). The samples were stained with an aqueous solution of phosphotungstic acid (1%, w/v) for approximately 2 min. A drop of each sample was then dipped on the carbon-coated copper grid, and the excess solution was absorbed using filter paper. The grid was allowed to air dry thoroughly, and the sample was observed and imaged.

***Encapsulation efficiency of CsA in nanomicelles***

A sample (100 μL) of CsA nanomicelle solution was added to 900 μL of methanol and vortexed for 2 min. Subsequently, a further 10-fold dilution with methanol was tested by HPLC. The encapsulation efficiency was expressed as the ratio of the detected to added drug amount.

***Physicochemical characterizations of CsA incorporated***

IR absorption spectrophotometry, DSC, and XRD measurements were used to test the physical state of CsA incorporated into the nanomicelles. These procedures are described in detail in the supporting information Fig.S1, Fig.S2, and Fig.S3.

***In vitro* CsA/Cou-6 leakage detection from nanomicelle**

The efficiency of Cou-6 as a marker for the nanomicelles was evaluated by measuring the leakage of CsA and Cou-6 from nanomicelles using a previously reported method^3^. SFM at pH 7.4 and pH 5.5 were used as the leakage media. First, 1mL of newly prepared nanomicelles containing 0.45 mg CsA and 0.05 mg Cou-6/mL was placed in a dialysis bag, sealed, and placed in 99 ml of a different medium. The container was continuously shaken at 100 rpm at 37 °C, and 1 mL of medium was aspirated at specific intervals for determination of CsA and Cou-6 leakage. A 1 mL volume of fresh medium was added to replace the amount removed for analysis. Based on the primary experiments and previous reports, this test procedure could maintain the sink condition to both CsA and Cou-6, and this dialysis method could be effectively used to test the leakage or in vitro release to CsA and Cou-6^4-6^.

**Quantitative determination of CsA in aqueous humor and corneas**

Aqueous humor samples were analyzed by mixing 100 µL aqueous humor with 800 µL of [acetonitrile](app:ds:acetonitrile), vortexing for 2 min, and centrifuging at 7,378g for 10 min. The corneas were assayed by weighing and homogenizing 50 mg tissue/1.0 mL [acetonitrile](app:ds:acetonitrile) and centrifuging at 7,378g for 10 min. Aliquots of the supernatants of all samples were filtered through 0.45 μm membranes and were then analyzed with an Acquity Ultra Performance liquid chromatography (UPLC)-Quattro Premier XE Tandem quadrupole mass spectrometer (UPLC-MS, Waters Corporation, Milford, MA, USA). CsD was chosen as the internal standard. This technique provided robustness, high specificity, and excellent sensitivity for drug measurements. The chromatographic conditions were as follows: A Waters XSELECT^TM^ HSS CN chromatographic column (2.1 mm×50 mm, 2.5 μm, Waters Corporation, Milford, MA, USA) was used for the separation; the mobile phase was a 90:10, and a v/v mixture of acetonitrile and 10 mmol/l ammonium acetate aq. was used at a flow rate of 0.2 mL/min. The UPLC analyses were performed at 30ºC. The mass spectrum was as follows: ESI^+^ was used as the ionization mode and multiple reaction monitoring (MRM) as the detection mode; the capillary voltage was 4 kV, and the extractor voltage was 4 V. The ion source temperature was 110ºC, the desolvation temperature was 350ºC, the desolvation gas flow rate was 550 L/h, the cone gas flow rate was 50 L/h, and the argon gas flow was 0.26 mL/min. The other MS parameters for the detection of CsA and CsD are listed in supporting information Table S2. All results were expressed as nanograms (ng) of CsA per gram (g) or milliliter (mL) of cornea or aqueous humor.

**SI Supplementary data**

Table S1 Regulators and their concentrations used in the mechanistic study.

|  | Concentration | Effect |
| --- | --- | --- |
| Hypertonic sucrose | 0.45M | Inhibitor of clathrin-mediated endocytosis by K^+^ depletion effect |
| Chlorpromazine | 6µg/mL | Specific inhibitor of clathrin mediated endocytosis |
| Chloroquine | 125µM | Disrupting endosomes and lysosomes, prevents endosome acidification and causes swelling to endosomes and lysosomes |
| Indomethacin | 100µM | Inhibitor of caveolar-mediated endocytosis |
| NaN_3_ | 0.10% | General inhibitor of endocytic processes |
| Nystatin | 10µg/mL | Inhibitor of lipid raft/caveolae dependent endocytosis by cholesterol sequestration effect |
| Methyl-β-Cyclodextrin (MβCD) | 10mM | Cholesterol depletion agent, Effective inhibitor of lipid raft/caveolae dependent endocytosis |
| Phloridzin | 200µM | Nontransportable competitive inhibitor, Sodium-glucose cotransporter inhibitor |
| Heparin | 100µg/mL | specific inhibitor of heparan sulfate proteoglycans(HSPGs) |
| Amiloride | 10µM | Specific inhibitor of macropinocytosis |

Table S2. MS parameters

| Compound | Parent ion  *m/z* | Fragment ions  *m/z* | Cone voltage  /V | Collision energy  /eV |
| --- | --- | --- | --- | --- |
| CsA | 1203.88 | 425.96 | 60 | 45 |
|  |  | 1186.56 | 60 | 26 |
| CsD | 1217.32 | 425.91 | 60 | 46 |
|  |  | 1199.68 | 60 | 25 |


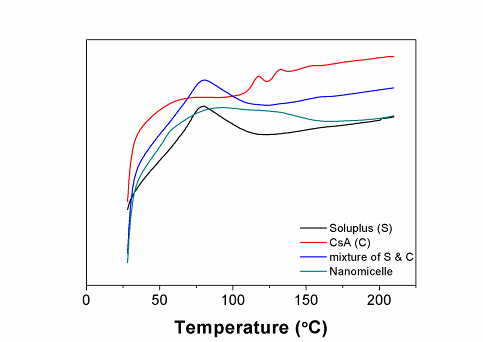


Figure S1 IR spectra for PVCL-PVA-PEG, CsA, a physical mixture of PVCL-PVA-PEG and CsA, and powdered CsA nanomicelles prepared by lyophilization were obtained using a VERTEX70 IR-spectrophotometer (BRUKER Corporation, Bremen, Germany). Potassium bromide discs containing the samples were prepared prior to the analysis. The PVCL-PVA-PEG thermogram displayed a thermal event at 80ºC, corresponding to the polymer glass transition temperature (Tg), while CsA exhibited a melting peak at 133ºC. The PVCL-PVA-PEG Tg value remained located at practically the same temperature for the physical mixture of PVCL-PVA-PEG and CsA, but the CsA melting peak was covered by the peak for PVCL-PVA-PEG. When the polymer was formulated in nanomicelles containing CsA, the peak corresponding to the CsA melting point disappeared, and the melting range showed that the polymer was significantly extended, indicating that CsA was dispersed within the nanomicelles in a non-crystalline state.


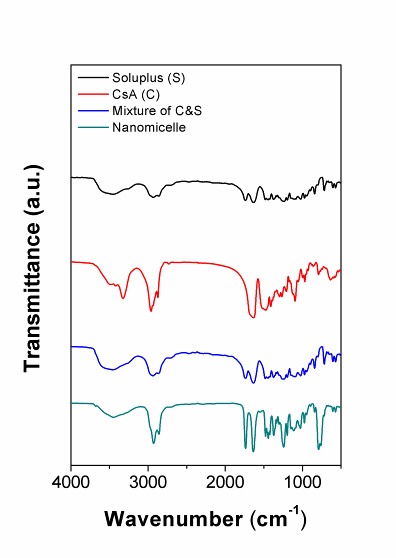


Figure S2 Thermal analysis was performed with a DSC204F1 differential scanning calorimeter (NETZSCH Group, Selb, Germany). For DSC measurements, aluminum pans were filled with samples weighing 5–10 mg, and the samples were heated from 28 to 200ºC at a rate of 10ºC/min in a nitrogen atmosphere (flow rate 100 mL/min). Under these conditions, DSC analysis data were obtained for PVCL-PVA-PEG, CsA, a physical mixture of PVCL-PVA-PEG and CsA, and CsA nanomicelle powder obtained by lyophilization. The CsA IR spectrum showed C=O, -CH3/-CH2-, and -C-O-C- stretching bands at 1600-1500 cm^−1^, 2930-2850 cm^−1^, and 1250 cm^−1^, respectively. The spectrum for PVCL-PVA-PEG was observed at 1470-1330 cm^−1^ and at 850-780 cm^−1^ for -CH3/-CH2- and for the phenyl stretching bands. Comparing the spectrum of freeze-dried CsA-loaded PVCL-PVA-PEG nanomicelles with that of the physical mixture of CsA and PVCL-PVA-PEG revealed no new absorption peaks, indicating the absence of any chemical reactions during the sample preparation procedures.


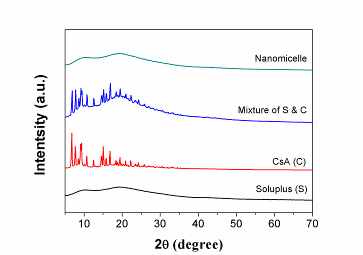


Figure S3 XRD analysis was performed with a Bruker D8 ADVANCE (Bruker Corporation, Germany). XRD data were obtained for PVCL-PVA-PEG, CsA, a physical mixture of PVCL-PVA-PEG and CsA, and CsA nanomicelle powder obtained by lyophilization. The diffraction pattern of CsA revealed several sharp, high-intensity peaks at the diffraction angles, suggesting that the drug existed as a crystalline material. However, the diffraction patterns of PVCL-PVA-PEG corresponded to the amorphous state. The patterns for the physical mixture of PVCL-PVA-PEG and for CsA showed all the typical bands of the polymer and CsA. In the nanomicelles of CsA-PVCL-PVA-PEG, the diffraction pattern was similar to that of the amorphous structure of PVCL-PVA-PEG. The sharp peaks of CsA were lost, and an amorphous structure was formed, suggesting the formation of amorphous structures. Lack of crystallinity is evidence of the CsA having been formulated into the nanomicelles.

**References**

1. Di Tommaso, C.*, et al.* Ocular biocompatibility of novel Cyclosporin A formulations based on methoxy poly(ethylene glycol)-hexylsubstituted poly(lactide) micelle carriers. *Int. J. Pharm.* **416**, 515-524 (2011).

2. Yu, H.*, et al.* Supersaturated polymeric micelles for oral cyclosporine A delivery. *Eur. J. Pharm. Biopharm.* **85**, 1325-1336 (2013).

3. He, B.*, et al.* The transport mechanisms of polymer nanoparticles in Caco-2 epithelial cells. *Biomaterials* **34**, 6082-6098 (2013).

4. Li, H., Xiao, Y., Niu, J., Chen, X. & Ping, Q. Preparation of a cationic nanoemulsome for intratumoral drug delivery and its enhancing effect on cellular uptake in vitro. *J. Nanosci. Nanotechnol.* **11**, 8547-8555 (2011).

5. Xia, H.*, et al.* Low molecular weight protamine-functionalized nanoparticles for drug delivery to the brain after intranasal administration. *Biomaterials* **32**, 9888-9898 (2011).

6. Wu, Y., Yao, J., Zhou, J. & Dahmani, F.Z. Enhanced and sustained topical ocular delivery of cyclosporine A in thermosensitive hyaluronic acid-based in situ forming microgels. *Int. J. Nanomedicine* **8**, 3587-3601 (2013).
